# Very-long-chain fatty acid metabolic capacity of 17-beta-hydroxysteroid dehydrogenase type 12 (HSD17B12) promotes replication of hepatitis C virus and related flaviviruses

**DOI:** 10.1038/s41598-020-61051-w

**Published:** 2020-03-04

**Authors:** Bassim Mohamed, Clément Mazeaud, Martin Baril, Donald Poirier, Aïssatou Aïcha Sow, Laurent Chatel-Chaix, Vladimir Titorenko, Daniel Lamarre

**Affiliations:** 1 Centre de Recherche du CHUM (CRCHUM) et Faculté de Médecine, Université de Montréal, Montréal, Canada; 2 Institut National de la Recherche Scientifique, Centre Armand-Frappier Santé Biotechnologie, Université du Québec, Laval, Canada; 3 Centre de recherche du CHU de Québec et Faculté de médecine, Université Laval, Québec, Canada; 4 Concordia University, Montréal, Canada; 5 Pharmacology Department, Medical Research Division, National Research Centre, Cairo, Egypt; 6 Department of Pathology, Case Western Reserve University, 2103 Cornell Road, Cleveland, Ohio 44106 USA

**Keywords:** Microbiology, Hepatitis C virus

## Abstract

*Flaviviridae* infections represent a major global health burden. By deciphering mechanistic aspects of hepatitis C virus (HCV)-host interactions, one could discover common strategy for inhibiting the replication of related flaviviruses. By elucidating the HCV interactome, we identified the 17-beta-hydroxysteroid dehydrogenase type 12 (HSD17B12) as a human hub of the very-long-chain fatty acid (VLCFA) synthesis pathway and core interactor. Here we show that HSD17B12 knockdown (KD) impairs HCV replication and reduces virion production. Mechanistically, depletion of HSD17B12 induces alterations in VLCFA-containing lipid species and a drastic reduction of lipid droplets (LDs) that play a critical role in virus assembly. Oleic acid supplementation rescues viral RNA replication and production of infectious particles in HSD17B12 depleted cells, supporting a specific role of VLCFA in HCV life cycle. Furthermore, the small-molecule HSD17B12 inhibitor, INH-12, significantly reduces replication and infectious particle production of HCV as well as dengue virus and Zika virus revealing a conserved requirement across *Flaviviridae* virus family. Overall, the data provide a strong rationale for the advanced evaluation of HSD17B12 inhibition as a promising broad-spectrum antiviral strategy for the treatment of *Flaviviridae* infections.

## Introduction

RNA viruses exploit basic cellular pathways and cytoplasmic organelles to achieve different stages of their replicative life cycle^1^. This is especially characteristic of *Flaviviridae*, *Coronaviridae* and *Picornaviridae* families of positive-strand RNA viruses that utilize cytoplasmic membranes for the formation of viral RNA replication and virion assembly compartments. Hepatitis C virus (HCV), a member of the *Flaviviridae* family, induce extensive endoplasmic reticulum (ER) membrane protrusions that generate double-membrane vesicles (DMVs) organized within a membranous web (MW), while other viruses such as flaviviruses dengue virus (DENV), Zika virus (ZIKV) and West Nile virus (WNV) use distinct ER invaginated vesicles as replication factories^2^. Mechanistically, the biogenesis of these virus-induced replication factories requires substantial structural changes to the ER membrane that involve membrane deformations, extensions and contractions to generate the appropriate architecture. As such, *de novo* synthesis of host lipids is crucial to the formation and functioning of these replication factories by facilitating membrane curvature and stimulating the activity of viral enzymes in the replication complex (for a review, see^3^). Furthermore, the proximity within the MW between these replication factories and lipid droplets (LDs), an organelle that is important in lipid storage and metabolism, contributes to the generation of infectious viral particles. Indeed, LDs are essential host components for the assembly of several *Flaviviridae* members^4–7^. As these viruses lack the appropriate enzymatic machinery to conduct their own lipid synthesis, they have evolved multiple mechanisms to hijack host fatty acid and lipid metabolic pathways important for the completion of their intracellular replication cycles^8–11^. Flaviviruses also trigger LDs lipophagy to drive virus production by exploiting the LD-localized type-III membrane AUP1 protein^12,13^.

Fatty acids are constituents of triglycerides, phospholipids and complex lipids, and their synthesis have long been identified as a requirement for the replication of many viruses including *Flaviviridae*^13–17^. They contribute to the structural integrity of membranes, energy production and storage, and generation of LDs. Saturated fatty acids up to 16 carbon atoms (C16) in length are synthesized in the cytosol by the multifunctional protein fatty acid synthase (FASN), which utilizes acetyl-CoA (C2:0-CoA), malonyl-CoA and NADPH to elongate fatty acids through a two-carbon increment process. Upon infection, the production of fatty acids and neutral lipids is often provided by an increase in FASN abundance and activity, which allow HCV propagation without transporting FASN to replication sites^16^. Others, like DENV, directly manipulate *de novo* fatty acid synthesis of palmitic acid (C16:0) by re-localization of FASN to these sites^13,16,18^. Moreover, antiviral effects have been reported with inhibitors of acetyl-CoA carboxylase 1 (ACC1) and FASN, which severely impede the replication of *Flaviviridae* family members and enteroviruses^13,16,18^. Fatty acid elongation to very-long-chain fatty acids (VLCFA, ≥C18) and regulation during *Flaviviridae* life cycle, however, are poorly understood. While fatty acids and derived lipids are used at different stages of viral life cycles, little is known about the modulation and requirement for *de novo* VLCFA synthesis to promote infection. The elongation of fatty acid beyond palmitic acid takes place at the ER membrane and involves multiple enzymes that act together as one physiological functional unit to catalyze four reactions in each two-carbon increment elongation cycle. These enzymes include elongation of very-long-chain fatty acid elongase subtypes 1 to 7 (ELOVL1–7) (1^st^ reaction), 17-beta-hydroxysteroid dehydrogenase type 12 (HSD17B12, also named DHB12) (2^nd^ reaction), very-long-chain (3 R)-3-hydroxyacyl-CoA dehydratase subtypes 1 to 4 (HACD 1–4) (3^rd^ reaction) and very-long-chain enoyl-CoA reductase (TECR) (4^th^ reaction).

HSD17B12 was first described as a key enzyme of steroid metabolism pathway^19^ and then identified as the human homolog of the yeast 3-ketoacyl-CoA reductase that catalyzes the second reaction in each VLCFA elongation cycle^20^. It interacts with all seven elongase ELOVL1–7 and of the 4 dehydratase HACD1–4 to generate the diversity among saturated, mono and polyunsaturated VLCFA species^21^. HSD17B12 is a metabolic hub of the VLCFA synthesis pathway^22^ that was identified in our HCV interactome screen as an interactor of the structural core protein^23^. Furthermore, HCV infection was recently reported to increase intracellular concentrations of VLCFA consistent with the engagement of intrahepatic LDs during viral assembly and accumulation of hepatic lipids during steatosis in infected patients^24^. Hence, we investigated the role of HSD17B12 during the replication of HCV and related flaviviruses. We showed that HSD17B12 knockdown (KD) impairs the ER-associated HCV RNA replication sites and severely reduces the number of cytoplasmic LDs. The KD of HSD17B12 result in significant inhibition of HCV, DENV and ZIKV infectious particle production, which is associated with a reduced abundance of phosphatidylethanolamine (PE), triglycerides (TG) and oleic acids (C18:1) in cells as revealed by shotgun lipidomics analysis. We further show that a rationally designed small-molecule steroid-based HSD17B12 inhibitor, INH-12, reduces the replication of HCV, DENV and ZIKV. The data support a contribution of *de novo* VLCFA synthesis for *Flaviviridae* virus infections and provide a strong rationale to explore the broad-spectrum antiviral potential of targeting HSD17B12 to treat and/or prevent RNA virus infections.

## Results

### HSD17B12 redistributes to replication and assembly sites during HCV infection

**Figure 1:**
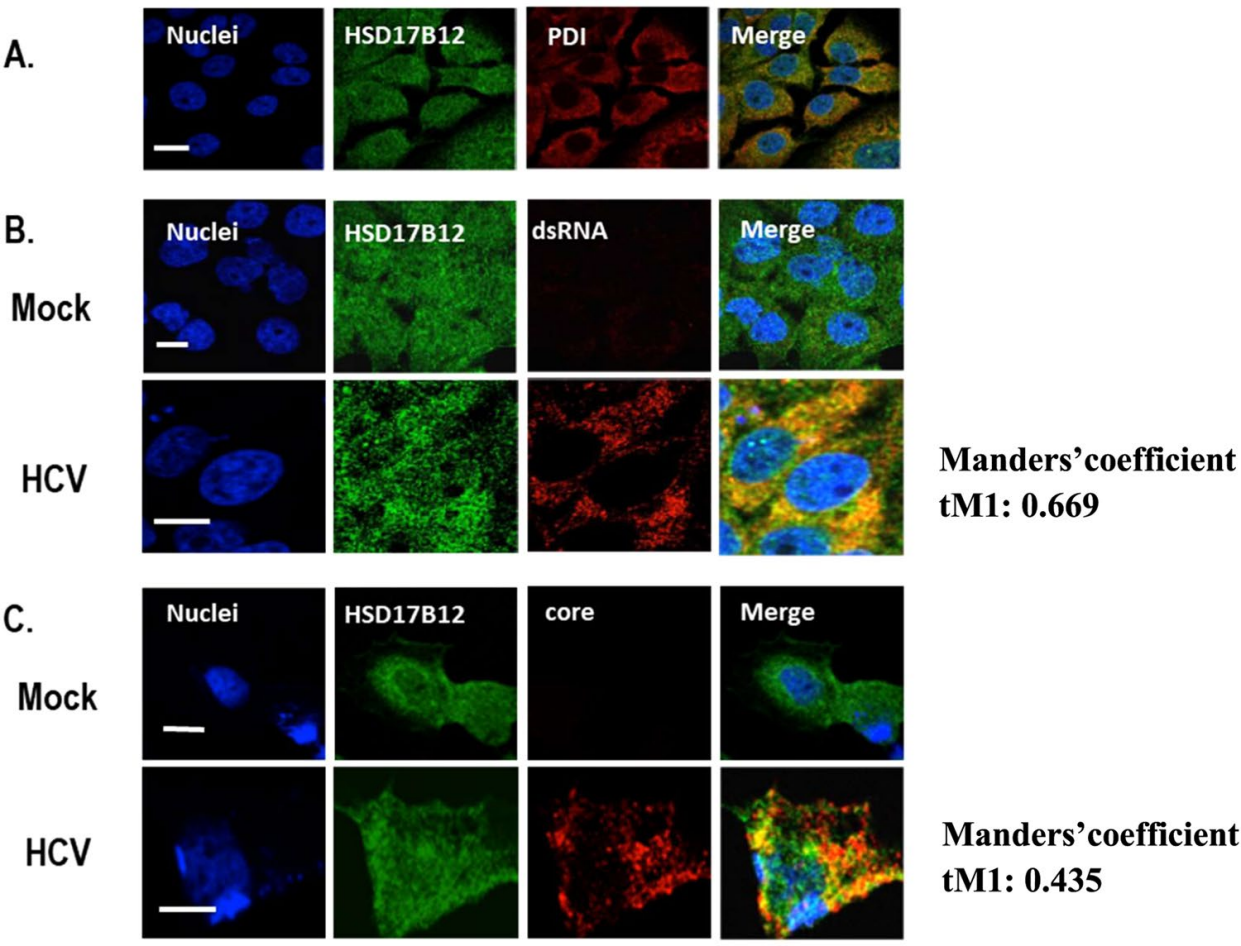
HSD17B12 overlaps replication and core-associated assembly sites of HCV infection. (**A**) Parental Huh7.5 cells were fixed with paraformaldehyde, permeabilized with 0.1% Triton X-100 and co-stained with anti-HSD17B12 and anti-PDI antibodies. (**B**) Huh7.5 cells and transfected with empty (Mock) or JFH-1 expressing DNA (HCV) plasmids. Four days later, cells were fixed and co-stained with anti-HSD17B12 and anti-dsRNA antibodies or (**C**) with anti-HSD17B12 and anti-core antibodies. Nuclei were stained with Hoechst. Fluorescent images were obtained using a confocal laser scanning microscope. Merged images are presented on the right column. Scale bars represent 20 µm. Manders’ colocalization values of images stacks were done using Coloc 2 (Fiji). The indicated tM1 Manders’ coefficient corresponds to the ratio of total signal from green channel (HSD17B12) that overlap with signal above a threshold intensity from red channel (dsRNA or core).

HSD17B12 was previously shown to interact with HCV core^23^ suggesting that HCV co-opts HSD17B12 functions during the infection. To further assess the cellular localization of HSD17B12, naïve and HCV-replicating Huh7.5 human hepatoma cells were imaged using confocal fluorescence microscopy (Figs. 1 and S1). HSD17B12 contains a C-terminal di-lysine motif that confers ER localization for type I membrane proteins, and previous study showed that the enzyme co-localizes with the ER resident protein calnexin^20^. We confirmed that HSD17B12 is an ER resident protein in Huh7.5 cells by the co-staining with protein disulfide isomerase (PDI) used as an ER marker (Fig. 1A). Upon HCV infection, we showed that a fraction of endogenous HSD17B12 overlaps viral replication and assembly sites as revealed by the co-staining with anti-HSD17B12 and anti-double-strand RNA (dsRNA) or anti-core antibodies in fluorescence microscopy, respectively. We measured threshold adjusted Mandel coefficients (tM1) values of 0.669 for dsRNA and 0.435 for core obtained from the quantitative colocalization analysis using Coloc 2 (Fiji’s plugin method) (Fig. 1B,C). Similar results are obtained with ectopic expression of a FLAG-tagged HSD17B12 fusion protein and detection with specific anti-FLAG antibodies in HCV-infected Huh7.5 cells (Fig. S1). The data support the hijacking of HSD17B12 at MW and further suggest that this promotes *de novo* synthesis of VLCFA and derived lipids for the benefit of HCV replication.

### HSD17B12 promotes HCV RNA replication and infectious particle production

**Figure 2:**
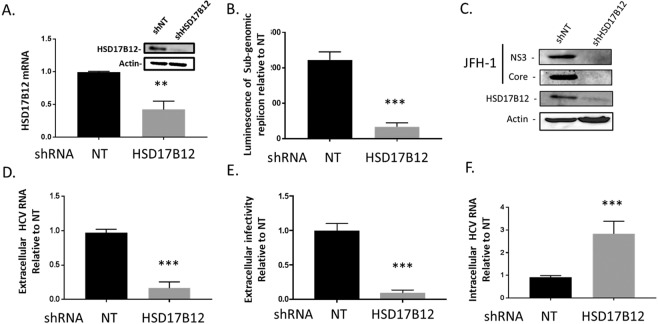
HSD17B12 KD decreases HCV replication and infectious viral particle production. (**A**) Huh7.5 cells transduced with lentiviruses expressing shNT or shHSD17B12 for 4 days were analyzed for HSD17B12 mRNA and protein levels. The effects of shNT (NT) and shHSD17B12 (HSD17B12) were determined on (**B**) reporter activity of Huh7 cells harboring an HCV subgenomic replicon (Huh7-Con1-Fluc) after 4 days, (**C**) NS3 and core protein levels of JFH-1-expressing Huh7.5 cells by western blot. Supernatants of cells transduced at day 0 with lentivirus expressing shNT or shHSD17B12 and transfected at day 2 with JFH-1 DNA were analyzed at day 6 for (**D**) extracellular HCV RNA levels and (**E**) extracellular infectivity by re-infecting naive Huh7.5 cells for 4 days and analysis of HCV RNA levels by qRT-PCR and (**F**) intracellular HCV RNA levels of JFH-1-expressing Huh7.5 cells by qRT-PCR. HCV RNA levels are normalized to actin RNA content and arbitrarily set to 1 for cells expressing shNT and infected with JFH-1. Values represent mean ± SD from the analysis of three experiments (n = 3). Unpaired student t test was used. P values < 0.05 (*), <0.01 (**) or <0.001 (***) are indicated in comparison with shRNA NT cells.

To get an insight into the role of HSD17B12 in virus infection, we first evaluated the impact of HSD17B12 knockdown (KD) on cell toxicity by transducing Huh7.5 cells with lentiviruses expressing short hairpin RNA (shRNA) specific to HSD17B12 or a non-targeting sequence (NT). Expression KD was validated at both mRNA and protein levels (Fig. 2A). We used Huh-7 cells stably expressing a firefly luciferase (Fluc) gene under the control of the EF1α housekeeping promoter to assess on- and off-target cytotoxic effects of shRNA-mediated HSD17B12 depletion (Fig. S2A). We showed that the luciferase signal was minimally disrupted indicating that HSD17B12 KD does not significantly affect general protein synthesis. In parallel, we demonstrated that HSD17B12 KD has no major effect on cell viability and proliferation using an Alamar Blue assay (Fig. S2B**)**. HSD17B12 KD was then used to assess its role in cells harboring an HCV replication-competent reporter subgenome (Huh7-Con1-Fluc) for which viral RNA replication occurs in the absence of virus entry and capsid assembly due to the lack of HCV structural proteins (Fig. 2B). We showed that KD of HSD17B12 in HCV replicon-containing Huh7-Con1-Fluc cells significantly decreased HCV RNA replication as monitored by luciferase assays performed at 4 days post-transduction compared to cells expressing shNT. The HCV protease inhibitor BILN2061^25^ was used as a positive control. To further investigate the contribution of HSD17B12 to viral replication and virion assembly, we analyzed cells harboring a full-length HCV infectious genome (Fig. 2C–F). We took advantage of the ribozyme-based full-length JFH-1 clone that upon DNA transfection of Huh7.5 generates the authentic ends of HCV RNA genome, and subsequently replicates to produce infectious particles that efficiently spread in the cell culture^26^. We had previously confirmed that such JFH-1 infectious system is replication- and assembly-competent using several known mutants and pharmacological inhibitors^27,28^. Under these conditions, we first determined the viral protein levels and the production of infectious particles at 4 days post-transfection (and at 6 days post-transduction). We detected a significant decrease of NS3 and core protein expression in HSD17B12 KD Huh7.5 cells when assessed by western blot analysis (Fig. 2C). As HSD17B12 interacts with core that co-localizes to LD, and that HSD17B12 KD decreases core protein levels, we postulated that HSD17B12 would significantly contribute to the production of infectious viral particles. To test this, we evaluated the release of extracellular HCV RNA and of supernatant-associated infectivity of HSD17B12 KD cells. We found a reduction of the extracellular HCV RNA levels using qRT-PCR analysis (Fig. 2D). In parallel, we demonstrated a significant decreased production of extracellular infectious particles by up to 9-fold as revealed by infection of naive Huh7.5 with the supernatants of HSD17B12 KD cells compared to control virus-producing cells expressing shNT (Fig. 2E). Similar results were obtained using the supernatants of HSD17B12 KD HepG2 cells, another liver cell line (Fig. S3). The assembly of HCV particles relies on the closed proximity between virus-induced replication structures and LDs. As such, we assessed the impact of a likely impaired viral assembly process on the intracellular HCV RNA levels. Surprisingly, we found that HSD17B12 KD led to a significant intracellular accumulation of HCV RNA compared to cells expressing shNT (Fig. 2F). Similar results were observed in HSD17B12-depleted HepG2 cells (Fig. S4). To rule out any effects on viral translation, the KD of HSD17B12 was investigated by co-expression of an HCV IRES-driven firefly luciferase (Fluc) and CMV-driven cap-dependent renilla luciferase (Rluc). No significant difference of HCV IRES-dependent translation was observed in HSD17B12 KD cells compared to cells expressing shNT when both reporter activities were monitored (Fig. S2C). While the low levels of viral proteins detected in HSD17B12 KD cells (Fig. 2C) should negatively impact HCV replication kinetics, the increased intracellular HCV RNA at day 4 possibly reflect an impaired nucleocapsid assembly promoting its retention. Altogether, the data strongly support a role of HSD17B12 during both replication and particle assembly steps of HCV life cycle.

### HSD17B12 contributes to a lipid metabolic environment for particle assembly

**Figure 3:**
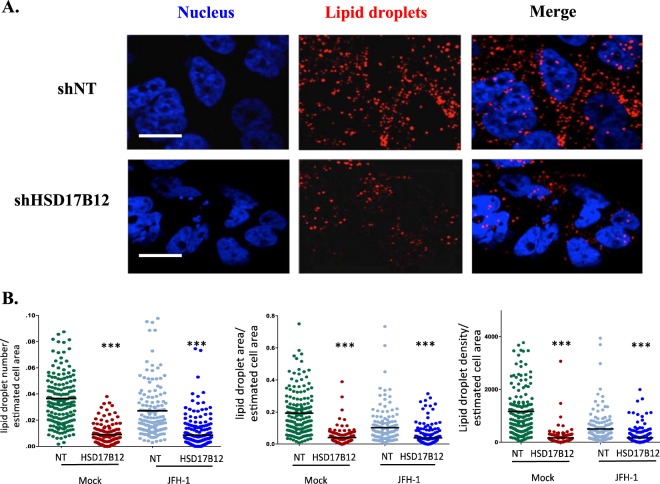
HSD17B12 KD reduces the number of cytoplasmic LDs. (**A**) Huh7.5 cells transduced with lentivirus-expressing shNT (NT) or shHSD17B12 (HSD17B12) are analyzed for the contents of LDs. Cells were fixed with paraformaldehyde, permeabilized with 0.1% Triton X-100 and stained with Lipidtox (LD) and Hoechst (nuclei). (**B**) Control and JFH-1 expressing cells were knocked-down or not for HSD17B12 expression and were imaged (7 field views) for each condition. The number, area and density of LDs per cell were quantified. Unpaired student t-test was used. P values < 0.001 (***) are indicated in comparison with cells expressing shNT. Scale bar = 20 µm.

**Figure 4:**
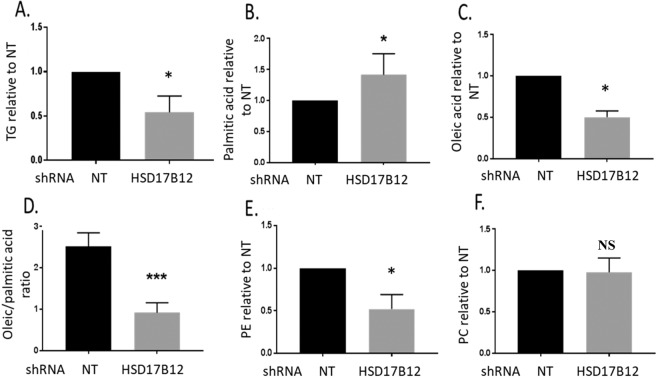
HSD17B12 KD induces alterations in cellular lipid metabolism. Huh7.5 parental cells transduced with lentiviruses expressing shNT (NT) or shHSD17B12 (HSD17B12) were used to determine the relative cellular abundance of (**A**) triglycerides (TG), (**B**) palmitic acid (C16:0), (**C**) oleic acid (C18:1), (**D**) oleic/palmitic (C18/C16) ratio, (**E**) phosphatidylethanolamine (PE) and (**F**) phosphatidylcholine (PC) among all detected lipid species by shotgun lipidomics analysis of whole cell extracts. The data of three experiments were pooled for the analysis (n = 3). Data were normalized to shNT control arbitrary value of 1 (except D). Non-parametric Mann Whitney test was used to compare each test condition with the control. P values < 0.05 (*) or non-significant (NS) are indicated in comparison with cells expressing shNT as control.

Lipid droplets (LDs) are essential to HCV assembly and their depletion decreased production of infectious virus particles^4,29^. LDs are composed mainly of triglycerides (TGs), which extensively incorporate VLCFA, especially stearic (C18:0) and oleic (C18:1) acids. As HSD17B12 is a key player of the VLCFA synthesis pathway, we postulated that its metabolic capacity contributes to the biogenesis and maintenance of LDs through requirement of newly synthesized TGs. To test this, we analyzed the abundance and size cytoplasmic LD organelles in HSD17B12-depleted cells by staining LDs with LipidTOX (Fig. 3A). Confocal imaging analysis revealed that HSD17B12 KD drastically decreases the relative number, areas and density of LDs in both uninfected and JFH-1-infected Huh7.5 cells (Fig. 3B). As compared to control cells expressing shNT, the reduced LDs in HSD17B12 KD cells can be explained by a decreased TG synthesis as a result of the deficiency in VLCFAs availability (≥C18). To further investigate whether HSD17B12 KD induces a lipid metabolic remodeling that disfavors HCV infection, we used a shotgun mass spectrometry lipidomics approach to measure fatty acid species and lipid families in whole cell extracts. We showed a significant decrease in the cellular contents of TGs of HSD17B12 KD cells compared to control cells (Fig. 4A). Furthermore, we found a decrease of the relative abundance of oleic acid (C18:1; HSD17B12-dependent) with concomitant increase of palmitic acid (C16:0; HSD17B12-independent) resulting in significant decreased of C18:C16 ratios that are expected from reduced HSD17B12 enzymatic activity (Fig. 4B–D). In line with these results, the supplementation of HSD17B12 KD cells with oleic acid rescued the cellular contents of LDs (Fig. S5). This demonstrates the requirement of HSD17B12 metabolic capacity for LD maintenance by *de novo* synthesis of VLCFA and TGs, as well as during HCV assembly on LDs. We also examined whether the reduced abundance of LDs is explained by an increased expression of catabolic lipid enzymes possibly due to the need of stored fatty acids for metabolic reactions. Interestingly, we detected striking increased mRNA levels of hormone sensitive lipase (LIPE) and phospholipase A2 (PLA2G1B) in HSD17B12 KD cells (Fig. S6). Given their role in lipolysis, these results suggest that reduced expression of HSD17B12 induces a feedback response that hydrolyses TGs of LDs. Such response, however, is unable to maintain cellular contents of oleic acid (C18:1) and TGs that are required for MW structure upon viral assembly. Interestingly, we also showed a significant decrease in the cellular contents of the phospholipid phosphatidylethanolamine (PE) (Fig. 4E), which was reported to be important for viral replication compartments of positive-strand RNA virus^30,31^, while no changes were observed for phosphatidylcholine (PC) species (Fig. 4F). The decreased VLCFA and lipid species of HSD17B12 KD cells contrast with the opposite metabolic needs of HCV-infected cells to synthesize ER-derived specialized MW structures and to expand LDs for virion assembly. Overall, our data strongly support the metabolic capacity of HSD17B12 in tailoring a lipid metabolic environment that favors HCV infection, and that its inhibition interferes with the replication and particle assembly processes by reduction of *de novo* VLCFA synthesis and lipid species.

### Depletion of HSD17B12 impairs HCV replication sites

**Figure 5:**
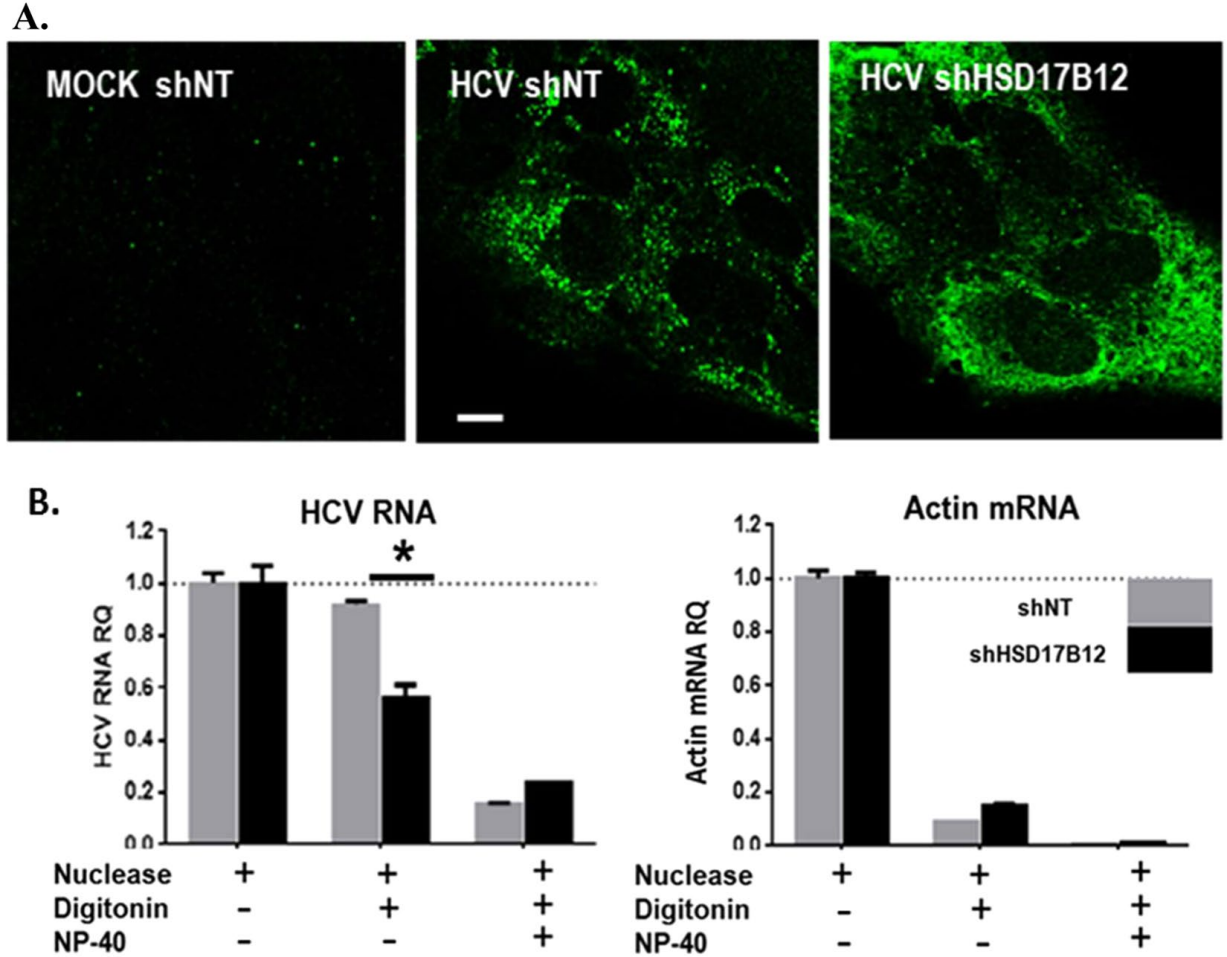
HSD17B12 KD induces dispersion and disruption of HCV replication sites. (**A**) Huh7.5 parental cells (MOCK shNT), infected with JFH-1 and transduced with lentiviruses expressing shNT (HCV shNT) or shHSD17B12 (HCV shHSD17B12) were stained with anti-dsRNA antibodies as makers of viral RNA replication sites and imaged by confocal microscopy. Results are representative of multiple field views. Scale bar = 10 µm (**B**) Huh7.5 cells transduced with lentiviruses expressing shNT or shHSD17B12 and transfected with JFH-1 DNA plasmids for four days are analyzed for RNA susceptibility to exogenous nucleases. Cells were treated with micrococcal nuclease, digitonin and/or NP40 as indicated by a+, and the relative abundance of HCV RNA (left panel) and actin mRNA (right panel) levels were determined by qRT-PCR analysis. RNA levels of cells treated with nuclease only are normalized to one. Values represent mean ± SD from the analysis of two experiments. Unpaired student t-test was used. P values < 0.05 (*) are indicated in comparison with shNT treatment.

HSD17B12 contributes to the synthesis of oleic acids that produce important concentration-dependent alterations of the lipid membrane structure^32^. In light of the altered metabolic capacity of HSD17B12 KD cells and reduced abundance of LDs (Fig. 3), significant biochemical, architectural and functional changes of HCV MW structures are expected. To test this, we first assessed the cellular distribution of dsRNA as a marker of viral RNA replication complexes in HSD17B12 KD cells by confocal microscopy (Fig. 5A). No anti-dsRNA antibody staining was observed in parental MOCK cells transduced with lentiviruses expressing shNT, validating the staining specificity of HCV RNA replication compartments. In JFH-1-replicating control cells expressing shNT, we showed a dot-like cytoplasmic staining with a well-defined distribution of replication sites into punctuated foci as previously reported^33^. In HSD17B12 KD cells, however, we observed a more diffuse staining with a wide-spread pattern of distribution suggesting a morphological alteration of the MW. The HCV-induced MW structures are known to be partially resistant to mild detergent treatment and to protect viral components from exogenous proteases and nucleases *in vitro*^34,35^. To assess the structural functionality of these diffuse replication sites of HSD17B12 KD cells, we performed an RNase protection assay on cell extracts using membrane permeabilizing detergent (digitonin) and exogenous nucleases as previously reported^4,36^ (Fig. 5B). As expected, the treatment of shNT-expressing control infected cells with both nuclease and digitonin resulted in less than 10% degradation of HCV RNA and 90% degradation of cytoplasmic actin mRNA used as control. However, more than 50% of HCV RNA was degraded upon depletion of HSD17B12. This is either due to a mislocalization of newly synthetized viral RNA within unprotected sites and/or to impaired replication sites most probably reflecting a modified membrane VLCFA composition. As a positive control, cells that were permeabilized with both digitonin and the stronger detergent NP40 leading to the almost complete degradation of HCV RNA upon incubation with exogenous nuclease treatment. Overall, the data demonstrate that HSD17B12 KD impairs HCV RNA replication sites.

### Oleate supplementation rescues HCV replication in HSD17B12KD cells

**Figure 6:**
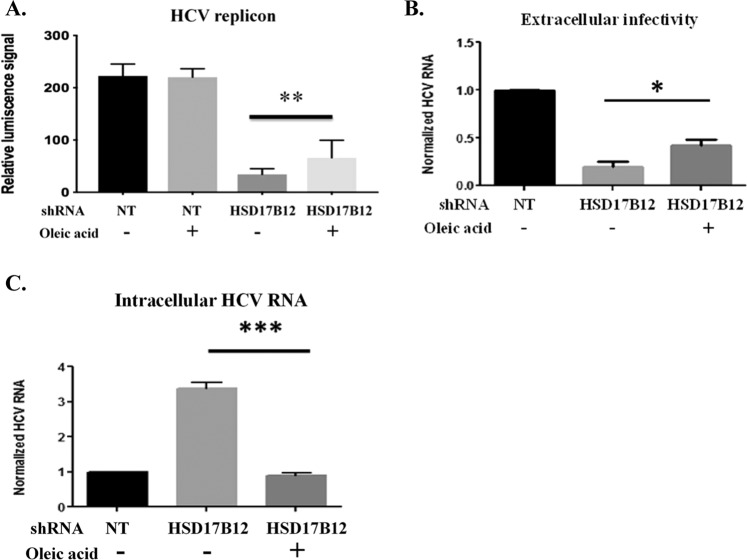
Oleic acid supplementation restores intracellular HCV RNA levels and partially rescues production of infectious particles in HSD17B12 KD cells. Huh7.5 cells transduced with lentiviruses expressing shNT (NT) or shHSD17B12 (HSD17B12) and infected with JFH-1 were used to evaluate the rescued effects of BSA-oleic acid treatment (20 µM) on (**A**) intracellular HCV RNA levels and (**B**) extracellular infectivity of supernatants upon re-infection of naive Huh-7.5 cells. HCV RNA levels were normalized with actin RNA contents and arbitrarily set to 1 for cells transduced with shNT and infected with JFH-1. Values represent mean ± SD from the analysis of three experiments. Unpaired student t test was used. P values, < 0.05 (*) or < 0.001 (***) are indicated in comparison with untreated shHSD17B12 transduced cells. (**C**) The effects of oleic acid treatment on viral replication were also determined in HSD17B12 KD cells stably expressing the HCV Con1B subgenomic replicon. Rescue experiments of HCV subgenomic replication were performed in HSD17B12 KD cells by addition of BSA-oleic acids (20 µM) to the cell culture media using the replication-dependent luciferase signals as readout. Data are representing analysis of 2 experiments (mean ± SD). One-way ANOVA with Tukey’s multiple comparisons test were used. P values < 0.01 (**) are indicated in comparison between untreated and oleic acid-treated shHSD17B12 transduced cells.

The requirement of oleic acid was previously reported for HCV replication^36^. Furthermore, HCV-infected cells were shown to contain a higher relative abundance of TGs and a strikingly increased utilization of C18 fatty acids, most prominently oleic acid^37^. As HSD17B12 KD decreases the cellular contents of oleic acid (Fig. 4C) and is associated with a decrease in viral replication and particle production correlating with an aberrant increased intracellular HCV RNA levels (Fig. 2), we hypothesized that supplementing HSD17B12 KD cells with oleic acid would restore HCV replication. Indeed, the addition of oleic acid (20 µM) to the cell culture medium of HSD17B12 KD cells partially rescued viral RNA replication of the sub-genomic replicon and JFH-1 extracellular particle production (Fig. 6A,B). Consistently, the oleic acid supplementation of JFH-1-expressing HSD17B12 KD cells restored viral RNA relative abundance to wild type levels (Fig. 6C). Together, the data support the requirement of oleic acid and other VLCFA species for HCV RNA replication, LD homeostasis and progeny particle assembly.

### HSD17B12 is required for ZIKV and DENV replication

**Figure 7:**
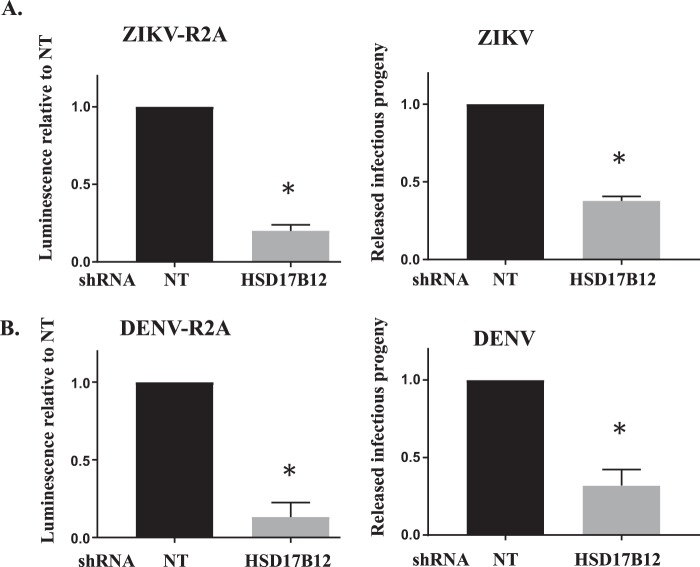
HSD17B12 KD decreases DENV and ZIKV replication and infectious viral particle production. Huh7.5 cells transduced with lentiviruses expressing shNT (NT) or shHSD17B12 (HSD17B12) were infected with (A - left panel) ZIKV FSS13025 strain and (B - left panel) DENV2 16681 strain both expressing a renilla luciferase (Rluc) gene. At 3 days post-infection, cells were analyzed for luminescence as readout of viral replication (left panels). To monitor viral particle production, Huh7.5 cells were infected with (A - right panel) wild type DENV 16681 strain and (B - right panel) wild type ZIKV H/PF/2013 strain. Supernatants were then analyzed for the content of infectious particle production by plaque assays. The luminescence signals and plaque forming units (PFU) of cells transduced with lentivirus-expressing shNT were arbitrarily set to 1. Values represent mean ± SD from the analysis of three experiments (n = 3). Non-parametric Mann Whitney test was used to compare each condition with the control. P values ≤ 0.05 (*) are indicated in comparison with shNT treatment.

**Figure 8:**
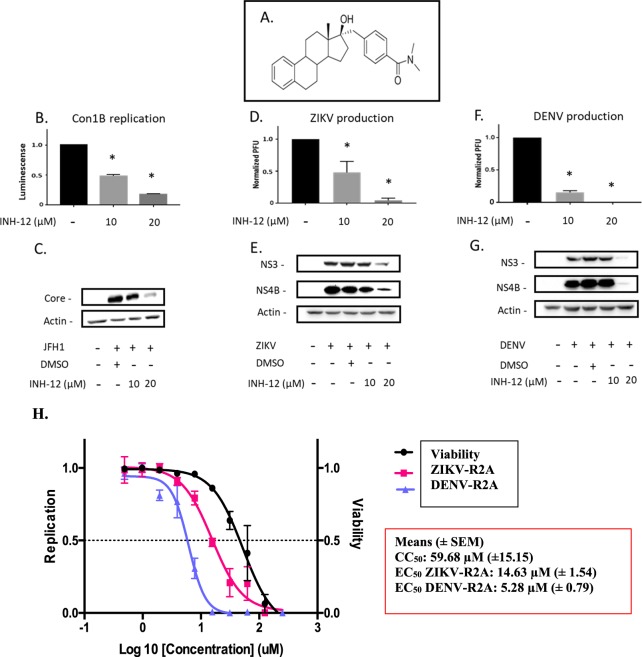
HSD17B12 inhibitor INH-12 blocks HCV, ZIKV and DENV replication. (**A**) Chemical structure of INH-12. (**B**) Huh-7 cells expressing an HCV subgenomic replicon Con1b luciferase were treated with 0 to 20 µM of INH-12 for 4 days. (**C**) Huh7.5 cells infected with JFH-1, (**D,E**) with ZIKV H/PF/2013 strain and (**F,G**) with DENV2 16681 strain were treated with 0 to 20 µM of INH-12 for 2 days. Infectious virus titers (**D**,**F**) were measured at a MOI = 0.05 for 3 days by plaque assays. The expression of viral proteins (**E**,**G**) was determined at a MOI = 3 at 2 days post-infection by western blot. Data were normalized to control arbitrary value of 1 (**D**–**F**). Non-parametric Mann Whitney test was used to compare each condition with the control. P values ≤ 0.05 (*) are indicated in comparison with DMSO control from analysis of 3 experiments (n = 3). (**H**) Representative EC_50_ and CC_50_ curves for INH-12 on Huh7.5 cells infected with DENV-R2A and ZIKV-R2A reporter viruses two days post-infection (MOI = 0.05) and table for mean values (±SEM for n = 4).

HCV and related flaviviruses manipulate *de novo* fatty acid synthesis of palmitic acid^13,16,18^, which serves as the main substrate for the synthesis of VLCFA. As very little is known about the requirement of VLCFA for flavivirus infection, we measured the replication of DENV and ZIKV in HSD17B12-depleted Huh7.5 cells (Fig. 7). HSD17B12 KD significantly decreased reporter DENV-R2A (strain 16681) and ZIKV-R2A (FSS13025 strain) replication as monitored by Rluc activities for both viruses. HSD17B12 KD also significantly reduced extracellular infectious titers of wild type DENV2 16681 and pathogenic ZIKV H/PF/2013 strains using plaque assays. We then evaluated the effects of INH-12, a small-molecule inhibitor of HSD17B12 on both HCV and flavivirus replication (Fig. 8). INH-12 is a steroid-based selective HSD17B12 inhibitor (Fig. 8A) synthesized at a greater than 99% purity, which has high solubility, high cell permeability and was derived from a series of compounds that are rarely cytotoxic at concentrations up to 50 µM^38^. The selective effects of INH-12 (C18 steroid derivative) on the enzymatic activity of HSD17B1, 5, 7 and 12 were reported^38^. INH-12 showed 70% inhibition of HSD17B12 activity to transform estrone (E1) into estradiol (E2) at 1 µM^39^. No inhibitory activity of C18 derivatives was observed against the C19 steroid metabolizing enzyme HSD17B3^38^. We first confirmed that INH-12 reduces viral replication of HCV replicon-containing cells (Figs. 8B and S7) and core protein expression of JFH-1 infected cells (Fig. 8C) at concentrations for which no appreciable cytotoxicity is observed (see Fig. 8H). The treatment of DENV- and ZIKV-infected Huh7.5 cells with INH-12 reduces the production of DENV infectious particles up to 3-log_10_-fold in multi-round infection assays (Fig. 8D,F), and block viral protein expression as monitored by western blotting in single-cycle infectious assays (Fig. 8E,G). We showed that INH-12 dose-dependently inhibits DENV and ZIKV with mean EC_50_ values of 5.3 µM and 14.6 µM, respectively, and demonstrated mean CC_50_ values of 59.7 µM in Huh7.5 cells (Fig. 8H). The data demonstrate the conserved requirement of HSD17B12 metabolic capacity for the replication of HCV and related flaviviruses DENV and ZIKV. They further validate pharmacologically the potential of HSD17B12 inhibition as an antiviral target with a pan-*Flaviviridae* spectrum.

## Discussion

The elongation of fatty acids to VLCFA and modulation of the pathway for virus-induced specialized membranous structures are poorly understood aspects of the biology of *Flaviviridae*. In this study, we characterized the requirement of HSD17B12 for HCV infection and its mode-of-action. This is a continuation of our HCV interactome study that identified HSD17B12 as a specific viral core interactor promoting HCV replication^23^. Interestingly, literature mining supports that HSD17B12 constitutes a hub protein that interacts with proteins of different RNA and DNA viruses^40–44^, which underline a potential broad-spectrum role of HSD17B12 during viral infections. HSD17B12 is an ER-bound keto-acyl reductase that catalyzes conversion between estrone and estradiol^19^, and was later reported to catalyze the second reaction of each VLCFA elongation cycle^20^. Our lipidomic analysis also confirms a function of HSD17B12 in VLCFA synthesis as revealed by the significant decreased of the oleic acid (C18.1)/palmitic acid (C16.0) ratio in HSD17B12 KD cells. In addition, a recent study described that VLCFA amounts are increased in HCV-infected cells^24^. Accordingly, patients with fatty liver display elevated levels of VLCFA in this organ^45^. This suggests an important role of the VLCFA synthesis pathway for HCV infection and pathogenesis. In this study, we provide strong and compelling evidence that the VLCFA metabolic capacity of HSD17B12 promotes the replication of HCV and flaviviruses DENV and ZIKV. First, HSD17B12 KD significantly decreases infectious particle production in Huh7.5 cells (Figs. 2, 7). Second, the inhibitor of HSD17B12, INH-12^38^, reduces viral RNA replication at concentrations for which no appreciable cytotoxicity is observed and dramatically reduces infectious particle production (>100-fold; Fig. 8). To our knowledge, this is the first report describing a proviral role of the metabolic capacity of HSD17B12-mediated VLCFA synthesis for several *Flaviviridae* members. Our data further highlight an additional antiviral mechanism-of-action other than FASN inhibitors^13,16,18^ through modulation of VLCFA metabolism with HSD17B12 inhibitors.

To mechanistically explore the contribution of HSD17B12 in promoting replication of pathogenic *Flaviviridae*, we focused our study on the replicative life cycle of HCV. We first provide evidence for the physical co-opting of a pool of ER resident HSD17B12 at virus-induced RNA replication and LD assembly sites (Figs. 1 and S1). The biogenesis of HCV MW (including DMVs and assembly complexes on LDs) likely requires specific lipid species and stoichiometry that provide the membrane biophysical characteristics needed for optimal viral genome replication and particle assembly processes^36,46,47^. FASN has the capacity to synthesize fatty acids containing up to 16 carbons such as palmitic acid, and was thought to be solely bringing the flux of fatty acids in proximity to replication and assembly compartments of flaviviruses^13,14,16,48^. However, FASN capacity to synthesize fatty acids is not sufficient to explain the enrichment of VLCFA at virus-induced specialized membrane sites^49,50^, since it requires the elongation enzymatic machineries. We now provide strong evidence of the requirement for *de novo* VLCFA synthesis with carbon chains ≥ C18 upon virus infection. Indeed, HSD17B12 KD blocks VLCFA elongation flux resulting in the expected increase of its substrate palmitic acid (C16:0) (Fig. 4). However, such increased palmitic acid levels are not enough to rescue HCV RNA replication (as reflected by the aberrant increased HCV RNA levels) and assembly (as reflected by the reduced extracellular infectious particles) (Figs. 2 and 7). Whether other elongation enzymes (i.e. ELOVL1–7, HACD1–4 and TECR) are hijacked to trigger *de novo* VLCFA synthesis will need further characterization of virus-induced specialized membrane compartments using proteomics and cell fractionation studies.

HSD17B12 KD decreased replication of subgenomic HCV RNA in absence of virus assembly but unexpectedly increased intracellular viral RNA levels of HCV-infected cells, which correlate with the reduction in oleic acid. We hypothesize that this HCV RNA increase might result from an accumulation of non-encapsidated genomes because of the loss of LD and assembly complexes. Since the reduction in membrane VLCFA such as oleic acid may impact the compartment integrity, whether the segregation of viral RNA synthesis at less defined virus-replicating sites contributes to the increased HCV RNA levels will need further ultrastructural studies and investigation. One explanation could rely on the intracellular accumulation of non-encapsidated HCV RNA close to unprotected viral replication sites. Indeed, because of the significant reduction of LD-based assembly platforms in HSD17B12 KD, the coordinated transfer of neosynthesized genomes between replication and assembly complexes would be dysregulated. Alternatively, such alteration of the membranous web may render the dsRNA more accessible by the anti-dsRNA antibodies during labelling, hence enhancing the signal in microscopy without necessarily reflecting increased dsRNA levels.

HSD17B12 KD reduces the number and size of LDs and decreases oleic acid and TG levels, which are the main components of LDs. The induction of lipolytic enzymes is observed and may reflect a cellular feedback requirement to increase cytoplasmic VLCFA involved in various vital metabolic processes. Interestingly, inhibition of lipolytic enzymes by core is rather observed upon HCV infection to maintain LD organelles^51,52^, impeding the activity of the hormone-sensitive lipase enzyme (LIPE)^53^ and the adipose triglyceride lipase (ATGL)^54^. In our experiments, however, we have not detected a significant increase in LDs upon HCV infection, in line with a recent report^37^, which may be due to the relatively short period of infection (4 days).

The LDs are crucial to several virus assembly processes^4–8^. But whether ZIKV and DENV share the same dependence on LDs is not clear. The two viruses exploit AUP1, a type-III membrane protein with dual localization signals for LDs and ER organelles^55^. AUP1 triggers LDs lipophagy, leading to the release of fatty acids for phospholipids synthesis and energy production. The AUP1−/− cells are resistant to ZIKV and DENV infection and virion production^12^. Nevertheless, LDs represent a compartment of the convergence of two important pathways involving proteins degradation (including HCV core protein): the ubiquitylation proteasomal degradation pathway and the lipophagy lysosomal degradation pathway. Defective LD compartment may change the signals that control those pathways leading to unbalanced exposure of the proteins in this compartment to degradative processes. Indeed, the targeting synthesis of lipids stored in LDs by triacsin C was previously shown to reduce stability of the viral core protein and suggested as an option for therapeutic intervention in treating chronic HCV infection^29^. Also, this model was investigated regarding ApoB protein, a protein that associates with HCV particle^56,57^. Thus, the metabolic capacity of HSD17B12 to maintain LDs most likely contributes to the production of HCV infectious particles. The decreased LDs in HSD17B12 KD cells is expected to lead to a rapid degradation of core protein as disrupting its localization to LDs leads to a destabilization of the protein^27,58–60^. While the supplementation of HSD17B12 KD cells with oleic acid rescued the cellular contents of LDs (Fig. S5), we did not observe a rescued in the decreased viral intracellular RNA levels and particles in ZIKV- and DENV-infected cells in contrast to HCV. This possibly reflects different requirements in lipid species (such as C18:0 stearic acid) for flavivirus replication. Along with the requirement of *de novo* VLCFA synthesis to maintain LDs, the inhibition of HSD17B12-mediated LD homeostasis as an essential platform for virus assembly and of HSD17B12-mediated VLCFA-derived lipids synthesis provide an antiviral mechanism-of-action for the significant reduction of HCV, DENV and ZIKV infectious particles. Finally, INH-12 is a selective inhibitor of HSD17B12 reductase activity, which supports a competitive mode-of-inhibition by occupation of the active site of the enzyme for its antiviral activity^19,38,39,61^. In summary, our study identifies HSD17B12 as a host co-factor involved in the replication of HCV and related flaviviruses DENV and ZIKV, and as a promising validated antiviral target. This study supports a conserved key role of *de novo* VLCFA synthesis in *Flaviviridae* infections and reveals the therapeutic potential of targeting HSD17B12 as a broad-spectrum antiviral approach.

## Methods

### Cell lines, antibodies and reagents

293 T, HeLa, VeroE6, Huh-7, Huh7.5 and HepG2 cell lines were cultured in Dulbecco’s modified Eagle’s medium (DMEM) containing 10% fetal bovine serum, 100 U/ml penicillin, 100 μg/ml streptomycin, 2 mM l-glutamine, and 1% nonessential amino acids (all from Wisent). Cells were transfected with linear (25-kDa) polyethylenimine (PEI; Polysciences) or jetPRIME (Polyplus-transfection, Invitrogen) as described by the manufacturer. Huh7 cells stably expressing a reporter Con1 subgenomic replicon (Huh7-Con1-Fluc) were maintained in complete DMEM with 500 μg/ml G418 (Multicell).

### Lentivirus production

293 T cells were transfected with PEI by using plasmids pRSV-REV, pMD2-VSVG, and pMDLg/pRRE and shRNA-encoding plasmid pLKO.1-puro (non-target and HSD17B12 TRCN0000027145, TRC 1 generation; Sigma-Aldrich). Lentiviruses were titrated using Hela cells as previously reported^28^. For gene silencing, cells were transduced shRNA-expressing lentiviruses at a multiplicity of infection (MOI) ≥ 2. Cells were collected for analysis according the design of different experiments.

### HCV infection assay

Huh7.5 cells were transfected with plasmid pEF/JFH-1-Rz/Neo^26^ by using JetPRIME (Polyplus-transfection, Invitrogen), and cell media was replaced after 4 h. At 4 days post-transfection, cells and culture media were collected. Virus-containing culture medium was cleared through a 0.45 μm filter and used to infect naive cells. Infected naive cells are collected after 4 days. Extracellular viral particles from cell culture supernatants were concentrated with 50 kDa Amicon- EMD Millipore) for RNA extraction and RT-PCR determination. We validated in previous studies viral replication of this system by the increased HCV RNA levels as a function of time that are blocked by HCV protease inhibitor BILN2061^25^. Furthermore, the validity of this system was extensively validated with both replication (NS5) and assembly (NS3, NS2 and p7) mutants^27,28^. When cells are transfected with the replication-defective JFH-1 DNA mutant (containing a point mutation in GDD motif to GND in the HCV NS5B polymerase) to determine the basal levels of HCV RNA (plasmid-dependent), we observed at least a 100-fold increase of intracellular HCV RNA levels with transfection of the wild type JFH-1 DNA at 4 days post-transfection and 6 days post-transduction.

### Cytotoxicity assays

Cells were cultured in transparent 96-well plates. Twenty microliters of 3-(4,5-dimethyl-2-thiazolyl)-2,5-diphenyl-2H-tetrazolium bromide (MTT) stock solution (5 mg/ml in PBS) was added to the cells and incubated for 1 h at 37 °C in the dark. Following the removal of the medium, cells were incubated at room temperature for 10 min with 150 μl of dimethyl sulfoxide (DMSO) containing 2 mM glycine, pH 11. Absorbance at 570 nm was read with a reference at 650 nm. For the Alamar Blue assay, cells were cultured in black 96-well plates. Ten microliters of Alamar Blue reagent (Invitrogen; diluted 1:4 in PBS) was added to the cells and following a 3 h incubation at 37 °C, fluorescence at 595 nm (excitation wavelength, 531 nm) was measured with an EnVision plate reader (PerkinElmer). A control plate with medium only (no cells) or Alamar Blue only was used to determine the background that was subtracted from the fluorescence value.

### qRT-PCR

Cells RNA was extracted using RNeasy Plus Kit. DNase treatment, reverse transcription and real-time PCRs were performed at the IRIC Genomic Core Facility with TaqMan-based assays. For HCV RNA detection, we used primers 5′-CATGGCGTTAGTATGAGTGTCG-3′ and 5′-GGTTCCGCAGACCACTATG-3′ and TaqMan-labeled probe CAGCCTCC (probe 75; no. 04688988001 from the Roche Universal Probe Library); for ZIKV H/PF/2013 strain, we used with primers 5′-GCCCTTCTGTTCACACCATT-3′ and 5′-CCACATTTGGGCGTAAGACT-3′ and for DENV2 16681 detection, we used the primers 5′-AGATGAACTGATTGGCCGGGC-3′ and 5′AGGTCTCTTCTGTGGAAATA-3′.

### Luciferase assays

Cells were washed once PBS. For firefly luciferase (Fluc) assays, 1 volume PBS and 1 volume of 2× luciferase buffer (100 mM Trizma acetate, 20 mM magnesium acetate, 2 mM EGTA, 1% Brij 58, 0.7% β-mercaptoethanol, 3.6 mM ATP, 45 μg/ml d-luciferin, pH 7.9) were added to the cells. Cells were incubated for 15 min at room temperature in the dark. For Renilla luciferase (Rluc) assays, 1 volume of PBS and 1 volume of 2 mM EDTA (pH 8)–5 μM coelenterazin (Nanolight) were added to the washed cells. Fluc and Rluc activities were measured with a luminescence counter (PerkinElmer).

### Antibodies

The following antibodies were used in this study: mouse anti-actin (EMD Milipore), mouse anti-HCV core (Affinity BioReagents), mouse anti-HCV NS3 (Abcam), mouse anti-Flag tag (Sigma), mouse anti-dsRNA (English Scientific Consulting), mouse anti-PDI (Stressgen), rabbit anti-HSD17B12 (Novus Biologicals), mouse anti-DENV NS3 (GeneTex; cross-reactive with ZIKV NS3) and rabbit DENV-NS4B (GeneTex; cross-reactive with ZIKV NS4B). Secondary antibodies coupled with horseradish peroxidase and Alexa Fluor were purchased from Bio-Rad and Invitrogen.

### Immunofluorescence analysis

Cells were grown on a coverslip in 6-well or 24-well plates, fixed, permeabilized and blocked as previously reported^28^. Following three rapid washes, cells were labeled for 2 h at room temperature with primary antibodies diluted in 5% BSA–0.02% sodium azide–PBS. Slides were washed three times in PBS and then probed with Alexa Fluor 488-, 594-conjugated secondary antibodies (Invitrogen) diluted 1:1000 in 5% BSA–0.02% sodium azide–PBS for 1 h in the dark. Cells were extensively washed and incubated with Hoechst dye (Invitrogen) at a final concentration of 1 μg/ml in PBS for 10 min. Following three rapid washes, the slides were mounted with 1,4-diazabicyclo [2.2.2] octane (Sigma-Aldrich) as an anti-fading agent. Labeled cells were then examined by confocal laser scanning microscopy (Leica TCS-SP5 MP) at the CRCHUM Imaging Core Facility or at the Centre Armand-Frappier Confocal Microscopy Facility. For lipid droplets staining, after fixation, permeabilization and blocking, the nuclei were stained with Hoechst. Following three rapid washes, cells were incubated for 1 h with HCS LipidTOX Deep Red (Invitrogen) diluted 1:1000 in PBS, immediately afterward cells were mounted with anti-fading agent. Labeled cells were then examined by Zeiss Observer microscopy. Data were analyzed by CRCHUM-Imaging Core Facility. For colocalization studies, Manders’coefficients are calculated by the proportional to the amount of fluorescence of the colocalizing pixels in each color channel. Values (tM1) range from 0 to 1 and express the fraction of intensity in the green channel (HSD17B12) that is located in pixels where there is above a threshold intensity in the red channel (dsRNA or core).

### RNase protection assay

The RNase protection assay was adapted from Lyn *et al*.^36^. Cells transduced with shNT and shHSD17B12 were transfected with JFH-1 plasmid for 4 days. Cells were washed once with cold buffer B (20 mM HEPES-KOH (pH 7.7), 110 mM potassium acetate, 2 mM magnesium acetate, 1 mM EGTA, and 2 mM dithiothreitol). For samples undergoing digitonin (Sigma-Aldrich, USA) treatment, buffer B containing 50 μg/ml of digitonin was added to cells for 5 min at 37 °C. The reaction was stopped by washing twice with cold buffer B. For samples treated with micrococcal nuclease (Sigma-Aldrich, USA) and/or NP-40 substitute (octyl-I-phenoxypoly-ethoxyethanol; Bioshop Canada Inc.), the cells were washed twice with buffer D (20 mM HEPES-KOH (pH 7.7), 110 mM potassium acetate, 2 mM magnesium acetate, 2 mM dithiothreitol, and 1 mM CaCl_2_) and treated with buffer D containing 0.1 unit/mL micrococcal nuclease, with or without, 0.45% NP-40 substitute for 15 min at 37 °C. Samples treated with 0.45% NP-40 substitute only were incubated for 10 min at 37 °C. Total RNA was extracted using the RNeasy Mini Kits (Qiagen) and treated with DNase. 250 ng of total RNA was reverse transcribed into cDNA, and equal amounts of cDNA processed for qRT-PCR at IRIC. The absolute amount of HCV RNA and actin were calculated.

### Lipidomic analysis

Tested cells were collected and pelleted. Cell pellets were kept on ice and re-suspend in 1 ml of nanopure water then were transferred to glass tubes. 20 µl of internal standard mix was added using the 25 µl glass syringe. Next, ~800 µl of glass beads were added using scoop device. Using a glass pipet, 3 ml of 17:1 CHCl_3_:MeOH was added to each tube. The tubes were vortexed at 4 °C (in the cold room) for 2 h and phases were separated by centrifugation at 3000 × g for 5 min. The lower organic phase was transferred to a clean 15 ml glass tube. The solvent of the organic phase tube was evaporated under nitrogen flow. Using a glass pipet 1.5 ml of 2:1 CHCl_3_:MeOH was added to the remaining aqueous phase. This phase was vortexed again at 4 °C (in the cold room) for 1 h. Phases were separated by centrifugation at 3000 g and combined with the organic phase from the 17:1 CHCl_3_:MeOH extract. The solvent was evaporated under nitrogen flow. Lipid film was dissolved in 150 µl of 2:1 CHCl_3_:MeOH and transferred to 2 ml glass vial with either Teflon or aluminum lined caps. Samples were stored at -20 °C until ready to be analyzed by mass spectrometry. Prior to infusing the samples in the mass spectrometer, samples were diluted in a 1:1 ratio using 2:1 CHCl_3_:MeOH + 0.1% NH_4_OH. Then samples are run through quantitative shotgun mass spectrometry using a high resolution Thermo Orbitrap Velos instrument. Data were analyzed as previously reported^62^. For free fatty acids (FFA), the relative abundance of specific fatty acid was calculated to total detected FFA. For TG, PC, PE, the relative abundance was calculated to total detected lipid species.

### HSD17B12 inhibitor INH-12 and antiviral assays

INH-12 (compound 97; Table 1 in Yannick Laplante, Master Thesis, U Laval, 2006, Chapter 4, page 121) was synthesized as reported^39^. The antiviral effects of INH-12 were determined on Huh7.5 cells transfected with JFH-1 plasmid and incubated with various concentrations (0–20 µM) of INH-12 for 4 days. Cells were analyzed by western blotting. HCV replicon containing cells were treated with INH-12 and analyzed by luminescence assay. For DENV and ZIKV replication, Huh7.5 cells were infected for 2 h at 37 °C at a multiplicity of infection (MOI) of 3 (i.e. 3 plaque forming units (PFU) per cell) in single round assays for western blot analysis, and at a MOI of 0.002 and 0.1 in multi-round assays for the luciferase replication assays and plaque assays, respectively. Virus inoculum was removed, cells were washed with PBS and incubated with various concentrations (0–20 µM) of INH-12. DMSO was used as control. Virus replication was analyzed by western blotting after 2 days, by luminescence assays after 2 days, and by plaque assay for virus titration at 3 days post-infection.

### Oleic acid supplementation

For rescue experiments, Huh7.5 cells were treated with oleic acids, which was prepared as previously described^28^. Briefly, 0.5 g of fatty-acid-free BSA (catalog number A6003; Sigma-Aldrich) was dissolved in 3.6 ml of 0.1 M Tris-Cl, pH 8. A 12.6-mg sample of oleic acid (catalog number O1008; Sigma-Aldrich) was transferred into clean tube and diluted with fatty-acid free BSA (catalog number A6003; Sigma-Aldrich) and then diluted in 3.6 ml of Tris-BSA buffer by gently shaking the solution until it was clear. The resulting concentration of this oleic acid stock was 12.5 mM.

### DENV/ZIKV titration by plaque assay and Rluc assays

Confluent monolayers of VeroE6 cells were infected with serial 10-fold dilutions of virus supernatants for 2 h at 37 °C. Two hours later, inoculum was removed and replaced with serum-free MEM (Gibco, Life Technologies) containing 1.5% carboxymethylcellulose (Sigma-Aldrich). Five days (ZIKV) or seven days (DENV) post-infection, cells were fixed for 2 h at room temperature with formaldehyde directly added to the medium to a final concentration of 5%. Fixed cells were washed extensively with water before being stained with a solution containing 1% crystal violet and 10% ethanol for 30 min. After rinsing with water, the number of plaques was counted at the appropriate dilution and virus titers were calculated. RNA replication of DENV and ZIKV reporter virus was determined 2 days post-infection by measuring the activity of virus-encoded renilla luciferase (Rluc) as previously described^63,64^. After lysis of the cells, coelenterazine (1.43 µM final concentration) was added and luminescence was measured.

### Statistical analysis

The statistical analysis was performed with the GraphPad Prism 7 software. Student *t-*test and non-parametric tests (Mann Whitney test and ANOVA with Tukey’s multiple comparisons test) were used, and P values ≤ 0.05 were considered significant.

## Supplementary information

Supplemental Figures 1-7.

## References

[1] Ravindran MS, Bagchi P, Cunningham CN, Tsai B (2016). Opportunistic intruders: how viruses orchestrate ER functions to infect cells. Nat. Rev. Microbiol..

[2] Chatel-Chaix L, Bartenschlager R (2014). Dengue virus- and hepatitis C virus-induced replication and assembly compartments: the enemy inside–caught in the web. J. Virology.

[3] Konan KV, Sanchez-Felipe L (2014). Lipids and RNA virus replication. Curr. Opin. Virol..

[4] Miyanari Y (2007). The lipid droplet is an important organelle for hepatitis C virus production. Nat. Cell. Biol..

[5] Samsa MM (2009). Dengue virus capsid protein usurps lipid droplets for viral particle formation. PLoS Pathog..

[6] Romero-Brey I (2012). Three-dimensional architecture and biogenesis of membrane structures associated with hepatitis C virus replication. PLoS Pathog..

[7] Shang Z, Song H, Shi Y, Qi J, Gao GF (2018). Crystal Structure of the Capsid Protein from Zika Virus. J. Mol. Biol..

[8] Heaton NS, Randall G (2010). Dengue virus-induced autophagy regulates lipid metabolism. Cell Host Microbe.

[9] Amako Y (2015). Hepatitis C virus attenuates mitochondrial lipid beta-oxidation by downregulating mitochondrial trifunctional-protein expression. J. Virol..

[10] Syed GH, Amako Y, Siddiqui A (2010). Hepatitis C virus hijacks host lipid metabolism. Trends Endocrinol. Metab..

[11] Bradley D (1991). Hepatitis C virus: buoyant density of the factor VIII-derived isolate in sucrose. J. Med. Virol..

[12] Zhang J (2018). Flaviviruses Exploit the Lipid Droplet Protein AUP1 to Trigger Lipophagy and Drive Virus Production. Cell Host Microbe.

[13] Heaton NS (2010). Dengue virus nonstructural protein 3 redistributes fatty acid synthase to sites of viral replication and increases cellular fatty acid synthesis. Proc. Natl Acad. Sci. USA.

[14] Martin-Acebes MA, Blazquez AB (2011). Jimenez de Oya, N., Escribano-Romero, E. & Saiz, J.C. West Nile virus replication requires fatty acid synthesis but is independent on phosphatidylinositol-4-phosphate lipids. PLoS One.

[15] Merino-Ramos T (2016). Modification of the Host Cell Lipid Metabolism Induced by Hypolipidemic Drugs Targeting the Acetyl Coenzyme A Carboxylase Impairs West Nile Virus Replication. Antimicrob. Agents Chemother..

[16] Nasheri N (2013). Modulation of fatty acid synthase enzyme activity and expression during hepatitis C virus replication. Chem. Biol..

[17] Villareal VA, Rodgers MA, Costello DA, Yang PL (2015). Targeting host lipid synthesis and metabolism to inhibit dengue and hepatitis C viruses. Antivir. Res..

[18] Nchoutmboube JA (2013). Increased long chain acyl-Coa synthetase activity and fatty acid import is linked to membrane synthesis for development of picornavirus replication organelles. PLoS Pathog..

[19] Luu-The V, Tremblay P, Labrie F (2006). Characterization of type 12 17beta-hydroxysteroid dehydrogenase, an isoform of type 3 17beta-hydroxysteroid dehydrogenase responsible for estradiol formation in women. Mol. Endocrinol..

[20] Moon YA, Horton JD (2003). Identification of two mammalian reductases involved in the two-carbon fatty acyl elongation cascade. J. Biol. Chem..

[21] Sassa T, Kihara A (2014). Metabolism of very long-chain Fatty acids: genes and pathophysiology. Biomol. Ther..

[22] Sakurai N (2006). Systemic distribution and tissue localizations of human 17beta-hydroxysteroid dehydrogenase type 12. J. Steroid Biochem. Mol. Biol..

[23] Germain MA (2014). Elucidating novel hepatitis C virus-host interactions using combined mass spectrometry and functional genomics approaches. Mol. Cell Proteom..

[24] Lupberger, J., *et al*. Combined Analysis of Metabolomes, Proteomes, and Transcriptomes of HCV-infected Cells and Liver to Identify Pathways Associated With Disease Development. *Gastroenterology* (2019). 10.1053/j.gastro.2019.04.003 PMC8318381 30978357

[25] Lamarre D (2003). An NS3 protease inhibitor with antiviral effects in humans infected with hepatitis C virus. Nature.

[26] Kato T (2007). Production of infectious hepatitis C virus of various genotypes in cell cultures. J. Virol..

[27] Chatel-Chaix L, Melancon P, Racine ME, Baril M, Lamarre D (2011). Y-box-binding protein 1 interacts with hepatitis C virus NS3/4A and influences the equilibrium between viral RNA replication and infectious particle production. J. Virol..

[28] Chatel-Chaix L (2013). A host YB-1 ribonucleoprotein complex is hijacked by hepatitis C virus for the control of NS3-dependent particle production. J. Virol..

[29] Liefhebber JM, Hague CV, Zhang Q, Wakelam MJ, McLauchlan J (2014). Modulation of triglyceride and cholesterol ester synthesis impairs assembly of infectious hepatitis C virus. J. Biol. Chem..

[30] Xu K, Nagy PD (2015). RNA virus replication depends on enrichment of phosphatidylethanolamine at replication sites in subcellular membranes. Proc. Natl Acad. Sci. USA.

[31] Xu K, Nagy PD (2016). Enrichment of Phosphatidylethanolamine in Viral Replication Compartments via Co-opting the Endosomal Rab5 Small GTPase by a Positive-Strand RNA Virus. PLoS Biol..

[32] Funari SS, Barcelo F, Escriba PV (2003). Effects of oleic acid and its congeners, elaidic and stearic acids, on the structural properties of phosphatidylethanolamine membranes. J. Lipid Res..

[33] Targett-Adams P, Boulant S, McLauchlan J (2008). Visualization of double-stranded RNA in cells supporting hepatitis C virus RNA replication. J. Virol..

[34] Wakita T (2005). Production of infectious hepatitis C virus in tissue culture from a cloned viral genome. Nat. Med..

[35] Zhong J (2005). Robust hepatitis C virus infection *in vitro*. Proc. Natl Acad. Sci. USA.

[36] Lyn RK (2014). Stearoyl-CoA desaturase inhibition blocks formation of hepatitis C virus-induced specialized membranes. Sci. Rep..

[37] Hofmann S (2018). Complex lipid metabolic remodeling is required for efficient hepatitis C virus replication. Biochim. Biophys. Acta Mol. Cell Biol. Lipids.

[38] Laplante Y, Rancourt C, Poirier D (2009). Relative involvement of three 17beta-hydroxysteroid dehydrogenases (types 1, 7 and 12) in the formation of estradiol in various breast cancer cell lines using selective inhibitors. Mol. Cell Endocrinol..

[39] Farhane S, Laplante Y, Poirier D (2011). Chemical synthesis, characterisation and biological evaluation of furanic-estradiol derivatives as inhibitors of 17beta-hydroxysteroid dehydrogenase type 1. Med. Chem..

[40] Jager S (2011). Global landscape of HIV-human protein complexes. Nature.

[41] Wu W (2012). The interactome of the human respiratory syncytial virus NS1 protein highlights multiple effects on host cell biology. J. Virol..

[42] Bradel-Tretheway BG (2011). Comprehensive proteomic analysis of influenza virus polymerase complex reveals a novel association with mitochondrial proteins and RNA polymerase accessory factors. J. Virol..

[43] Komarova AV (2011). Proteomic analysis of virus-host interactions in an infectious context using recombinant viruses. Mol. Cell Proteom..

[44] Rozenblatt-Rosen O (2012). Interpreting cancer genomes using systematic host network perturbations by tumour virus proteins. Nature.

[45] Farrell GC, Haczeyni F, Chitturi S (2018). Pathogenesis of NASH: How Metabolic Complications of Overnutrition Favour Lipotoxicity and Pro-Inflammatory Fatty Liver Disease. Adv. Exp. Med. Biol..

[46] Nguyen LN (2014). Stearoyl coenzyme A desaturase 1 is associated with hepatitis C virus replication complex and regulates viral replication. J. Virol..

[47] Lee WM, Ishikawa M, Ahlquist P (2001). Mutation of host delta9 fatty acid desaturase inhibits brome mosaic virus RNA replication between template recognition and RNA synthesis. J. Virol..

[48] Yang W (2008). Fatty acid synthase is up-regulated during hepatitis C virus infection and regulates hepatitis C virus entry and production. Hepatology.

[49] Berger KL, Kelly SM, Jordan TX, Tartell MA, Randall G (2011). Hepatitis C virus stimulates the phosphatidylinositol 4-kinase III alpha-dependent phosphatidylinositol 4-phosphate production that is essential for its replication. J. Virol..

[50] Perera R (2012). Dengue virus infection perturbs lipid homeostasis in infected mosquito cells. PLoS Pathog..

[51] Filipe A, McLauchlan J (2015). Hepatitis C virus and lipid droplets: finding a niche. Trends Mol. Med..

[52] Harris C, Herker E, Farese RV, Ott M (2011). Hepatitis C virus core protein decreases lipid droplet turnover: a mechanism for core-induced steatosis. J. Biol. Chem..

[53] Bose SK (2014). Forkhead box transcription factor regulation and lipid accumulation by hepatitis C virus. J. Virol..

[54] Camus G (2014). The hepatitis C virus core protein inhibits adipose triglyceride lipase (ATGL)-mediated lipid mobilization and enhances the ATGL interaction with comparative gene identification 58 (CGI-58) and lipid droplets. J. Biol. Chem..

[55] Klemm EJ, Spooner E, Ploegh HL (2011). Dual role of ancient ubiquitous protein 1 (AUP1) in lipid droplet accumulation and endoplasmic reticulum (ER) protein quality control. J. Biol. Chem..

[56] Fujimoto T, Ohsaki Y (2006). Proteasomal and autophagic pathways converge on lipid droplets. Autophagy.

[57] Ohsaki Y, Cheng J, Fujita A, Tokumoto T, Fujimoto T (2006). Cytoplasmic lipid droplets are sites of convergence of proteasomal and autophagic degradation of apolipoprotein B. Mol. Biol. Cell.

[58] Hope RG, McLauchlan J (2000). Sequence motifs required for lipid droplet association and protein stability are unique to the hepatitis C virus core protein. J. Gen. Virol..

[59] Vauloup-Fellous C (2006). Signal peptide peptidase-catalyzed cleavage of hepatitis C virus core protein is dispensable for virus budding but destabilizes the viral capsid. J. Biol. Chem..

[60] Boulant S, Vanbelle C, Ebel C, Penin F, Lavergne JP (2005). Hepatitis C virus core protein is a dimeric alpha-helical protein exhibiting membrane protein features. J. Virol..

[61] Laplante, Y. *In vitro* evaluation of inhibitors of 17beta-hydroxysteroid dehydrogenases type 1, 3, 12. Master thesis, Chapter 4, University of Laval, Quebec, Canada (2006).

[62] Richard VR, Bourque SD, Titorenko VI (2014). Metabolomic and lipidomic analyses of chronologically aging yeast. Methods Mol. Biol..

[63] Shan C (2016). An Infectious cDNA Clone of Zika Virus to Study Viral Virulence, Mosquito Transmission, and Antiviral Inhibitors. Cell Host Microbe.

[64] Kumar A (2013). Nuclear localization of dengue virus nonstructural protein 5 does not strictly correlate with efficient viral RNA replication and inhibition of type I interferon signaling. J. Virol..

