# Supplementary material for: Very-long-chain fatty acid metabolic capacity of 17-beta-hydroxysteroid dehydrogenase type 12 (HSD17B12) promotes replication of hepatitis C virus and related flaviviruses
Source: Sci Rep. 2020 Mar 4;10:4040. doi: 10.1038/s41598-020-61051-w (PMC7055353; doi:10.1038/s41598-020-61051-w)
Supplement: Supplementary file 1 — Supplemental Figures 1-7. [file 41598_2020_61051_MOESM1_ESM.pdf]

**SUPPLEMENTAL INFORMATION**

**Very-long-chain fatty acid metabolic capacity of 17-beta-hydroxysteroid dehydrogenase type 12 (HSD17B12) promotes replication of hepatitis C virus and related flaviviruses.** Bassim Mohamed, Clément Mazeaud, Martin Baril, Donald Poirier, Aïssatou Aïcha Sow, Laurent Chatel-Chaix, Vladimir Titorenko and Daniel Lamarre.

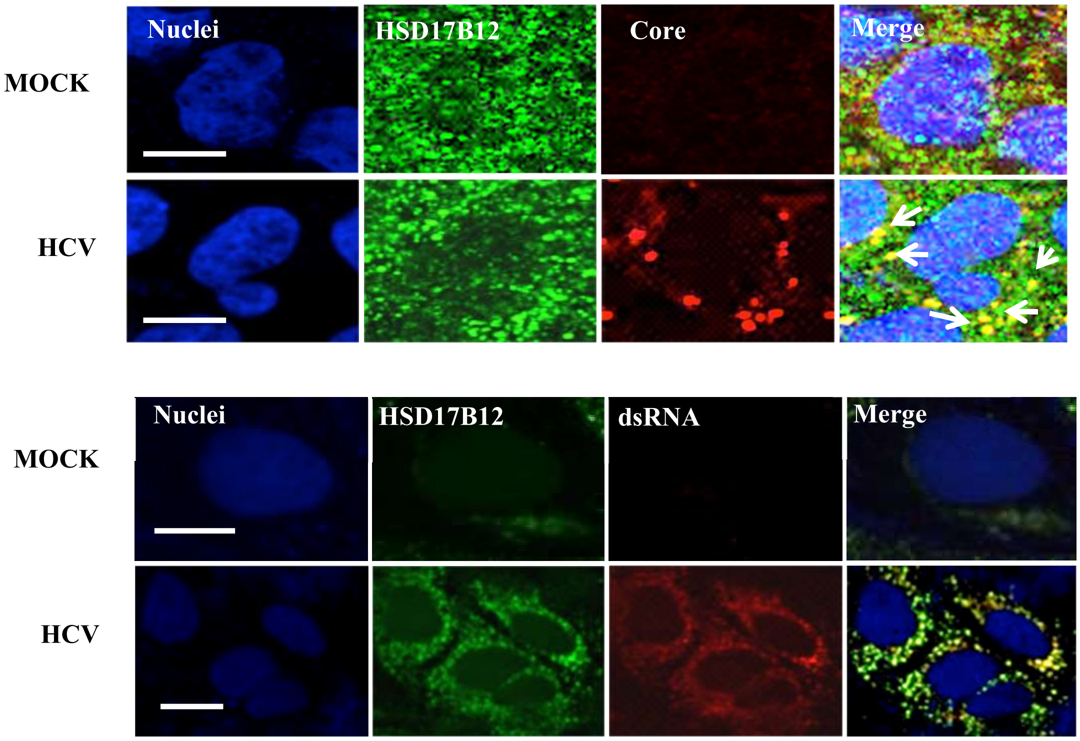


**Figure S1. HSD17B12 overlaps with HCV RNA replication and assembly sites.** Huh7.5 parental (Mock) and JFH-1-infected cells (HCV) were transfected with an N-terminal FLAG-tagged HSD17B12 fusion protein expressing vector. Cells were fixed with paraformaldehyde, permeabilized with 0.1% Triton X-100 and stained with anti-FLAG, anti-core and anti-dsRNA antibodies. Nuclei were stained with Hoechst. Images were obtained using a confocal laser scanning microscope. Co-localization is indicated by white arrows on merged images from the right column. Scale bars represent 20 μm.

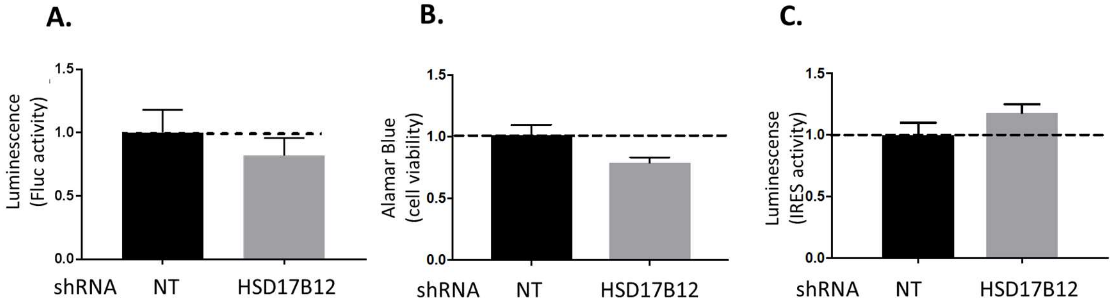


**Figure S2. HSD17B12 KD has no effect on protein translation, cell survival and HCV IRES-mediated translation.** Huh7 cells transduced with lentiviruses expressing shNT (NT) and shHSD17B12 (HSD17B12) were analyzed (A) for expression of a luciferase gene under an EF1-alpha promoter to assess general protein translation (B) for cell survival using an Alamar Blue assay and (C) for expression of HCV IRES-driven firefly luciferase normalized to CMV-driven renilla luciferase as a cap-dependent translation control. Error bars represent standard deviations from two experiments. No major differences were observed in all conditions.

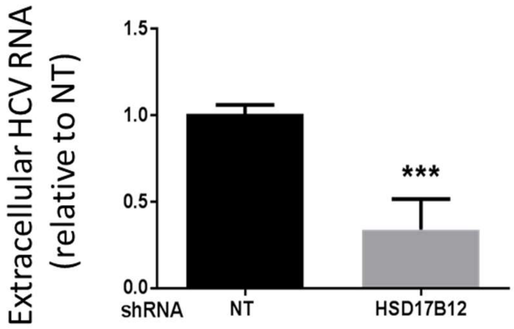


**Figure S3. HSD17B12 KD decreases extracellular HCV RNA levels of HepG2 infected cells.** HepG2 cells transduced with lentiviruses expressing shNT (NT) or shHSD17B12 (HSD17B12) were transfected with the JFH-1-expressing DNA plasmid for 4 days. Cell supernatants were analyzed for the extracellular HCV RNA levels using qRT-PCR and arbitrarily set to 1 for cells transduced with lentivirus expressing shNT. Error bars represent standard deviations from 3 biological replicates. P values < 0.001 (***) are indicated in comparison with shRNA NT treatment.

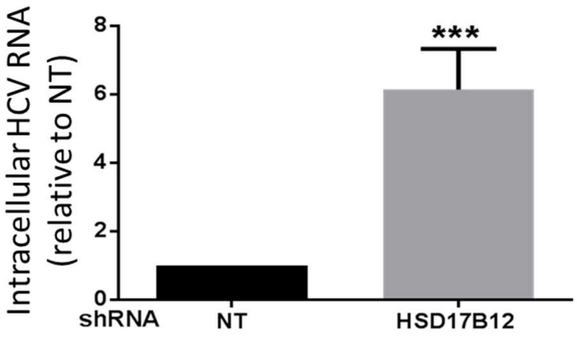


**Figure S4. HSD17B12 KD increases intracellular HCV RNA levels of HepG2-infected cells**. HepG2 cells transduced with lentiviruses expressing shNT (NT) and shHSD17B12 (HSD17B12) were transfected with the JFH-1-expressing DNA plasmid for 4 days. Cell extracts were then analyzed for the intracellular HCV RNA levels using qRT-PCR, normalized with actin RNA content and arbitrarily set to 1 for cells transduced with lentivirus expressing shNT. Error bars represent standard deviations from 3 biological replicates. P values < 0.001 (***) are indicated in comparison with shRNA NT treatment.

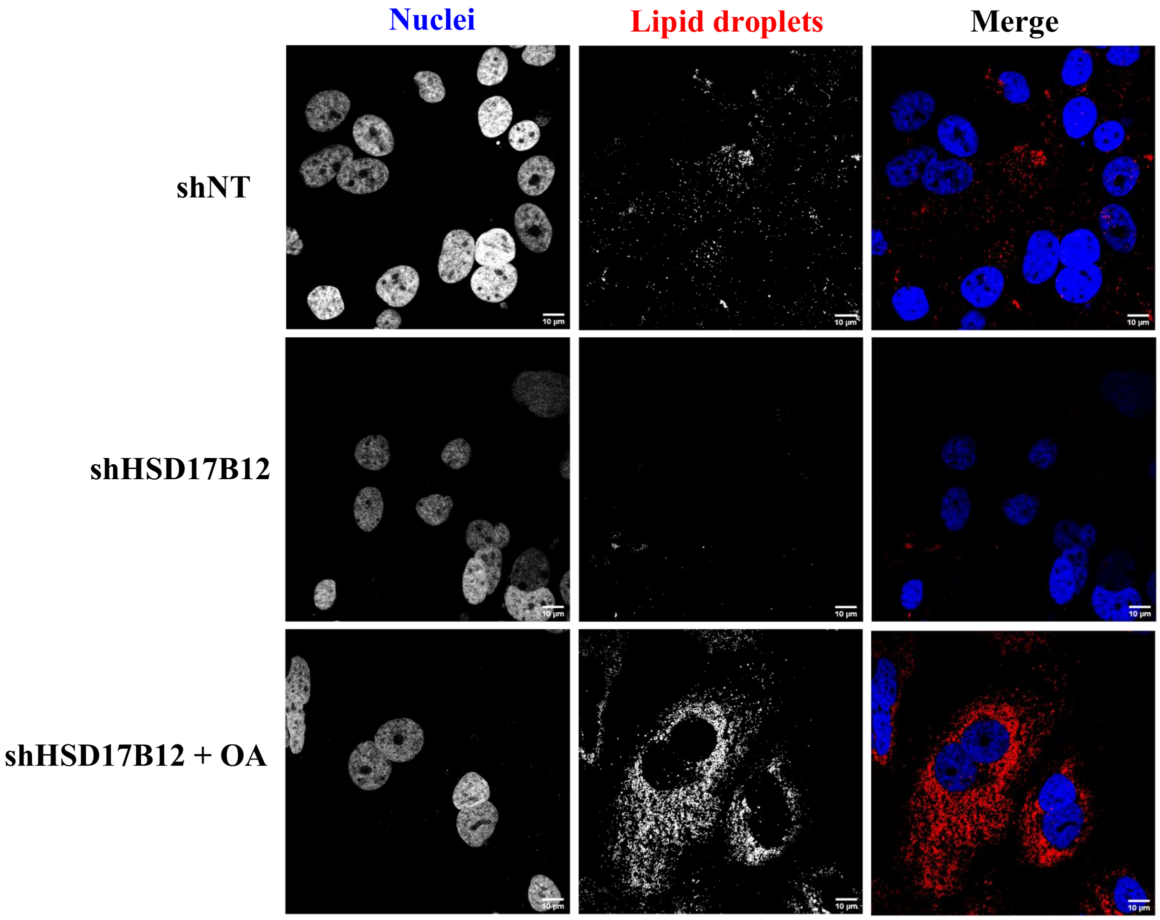


**Figure S5. Oleic acid preparation rescues lipid droplets in HSD17B12 KD Huh7.5 cells.** Three Huh7.5 cells conditions were prepared. A control condition transduced with shNT, and a test condition transduced with shHSD17B12, in addition to another test condition transduced with shHSD17B12 and treated with oleic acid preparation (50 µM). Five days post-transduction and 4 day-treatment with oleic acid, cells were stained with Lipidtox (LDs) and DAPI (nuclei) and were examined by fluorescence microscope for lipid droplets abundance. Scale bar = 10 µm.

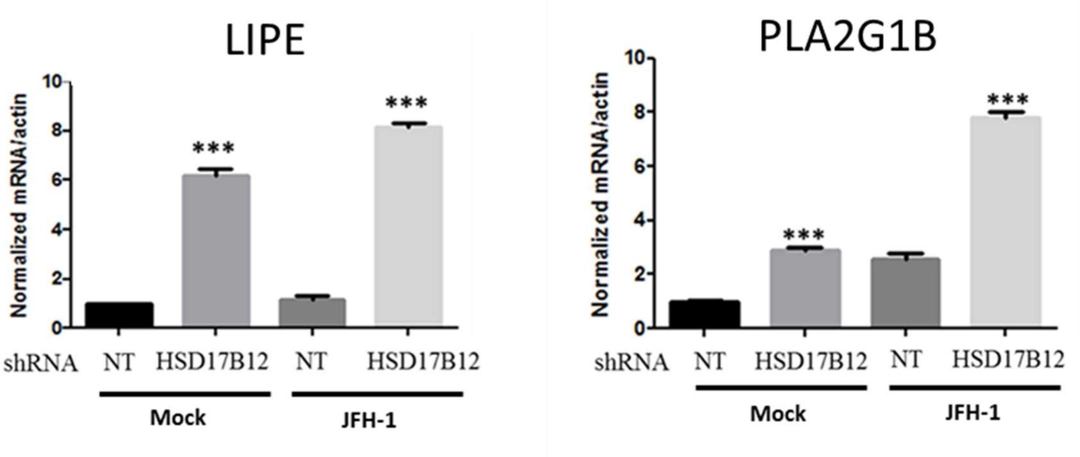


**Figure S6. HSD17B12 KD increases expression of lipolysis genes.** Huh7.5 cells transduced with lentiviruses expressing shNT (NT) or shHSD17B12 (HSD17B12), either uninfected (Mock) or transfected with JFH-1 DNA for four days are analyzed for mRNA levels of hormone sensitive lipase (LIPE) and phospholipase A2 (PLA2G1B) by RT-qPCR. Levels are normalized with actin RNA content and arbitrarily set to 1 for Mock NT cells. Error bars represent standard deviations from 3 biological replicates. P values < 0.001 (***) are indicated in comparison with shRNA NT treatment.

HCV Con1B-Fluc replication

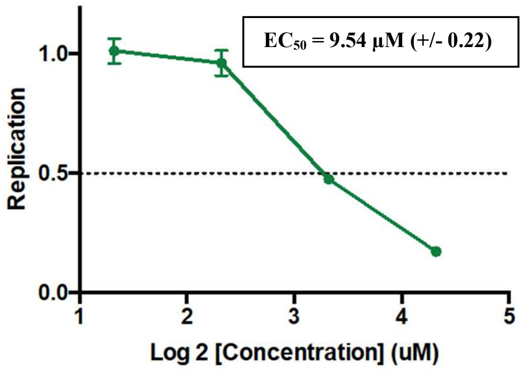


**Figure S7. HSD17B12 inhibitor INH-12 decreases HCV subgenomic replication.** The antiviral effects of treatment with various concentrations of small-molecule HSD17B12 inhibitor INH-12 were determined on HCV Con1b replicon-containing Huh7 cells using luciferase assays. INH-12 mean inhibitory concentration EC50 (+/- SEM) is indicated.
